# *QuickStats*: Percentage* of Persons of All Ages Who Had a Medically Attended Injury During the Past 3 Months,^†^ by Age Group — National Health Interview Survey,^§^ 2015–2017

**DOI:** 10.15585/mmwr.mm6805a7

**Published:** 2019-02-08

**Figure:**
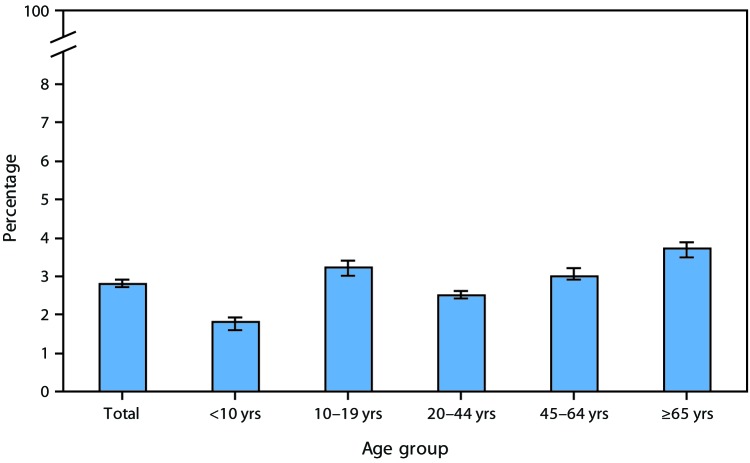
During 2015–2017, 2.8% of persons of all ages had a medically attended injury in the past 3 months, and this varied by age. The percentage who had a medically attended injury increased from 1.8% among those aged <10 years to 3.2% among those aged 10–19 years, declined to 2.5% among those aged 20–44 years, and then increased to 3.0% among those aged 45–64 years and to 3.7% among those aged ≥65 years.

**Source:** National Health Interview Survey, 2015–2017. https://www.cdc.gov/nchs/nhis.htm.

For more information on this topic, CDC recommends the following link: https://www.cdc.gov/injury/.

